# Effect of AAV-mediated overexpression of ATF5 and downstream targets of an integrated stress response in murine skeletal muscle

**DOI:** 10.1038/s41598-021-99432-4

**Published:** 2021-10-05

**Authors:** Madelaine C. Brearley-Sholto, David M. Loczenski-Brown, Sarah Jones, Zoe C. T. R. Daniel, Francis J. P. Ebling, Tim Parr, John M. Brameld

**Affiliations:** 1 School of Biosciences, University of Nottingham, Sutton Bonington Campus, Loughborough, LE12 5RD UK; 2 School of Life Sciences, University of Nottingham Medical School, Nottingham, NG7 2UH UK; 3 Present Address: Departments of Biological Chemistry and Medicine, University of California, Los Angeles, CA USA; 4 Present Address: Sygnature Discovery Limited, BioCity, Nottingham, NG1 1GR Nottinghamshire UK; 5 Present Address: School of Psychology, University of Nottingham, University Park, NG7 2RD UK

**Keywords:** Metabolism, Gene delivery, Reverse transcription polymerase chain reaction, Animal physiology

## Abstract

We previously reported that growth promoter-induced skeletal muscle hypertrophy co-ordinately upregulated expression of genes associated with an integrated stress response (ISR), as well as potential ISR regulators. We therefore used Adeno-Associated Virus (AAV)-mediated overexpression of these genes, individually or in combination, in mouse skeletal muscle to test whether they induced muscle hypertrophy. AAV of each target gene was injected into mouse *Tibialis anterior* (TA) and effects on skeletal muscle growth determined 28 days later. Individually, AAV constructs for Arginase-2 (*Arg2*) and Activating transcription factor-5 (*Atf5*) reduced hindlimb muscle weights and upregulated expression of genes associated with an ISR. AAV-*Atf5* also decreased Myosin heavy chain (MyHC)-IIB mRNA, but increased MyHC-IIA and isocitrate dehydrogenase-2 (*Idh2*) mRNA, suggesting ATF5 is a novel transcriptional regulator of *Idh2*. AAV-*Atf5* reduced the size of both TA oxidative and glycolytic fibres, without affecting fibre-type proportions, whereas *Atf5* combined with *Cebpg* (CCAAT enhancer binding protein-gamma) only reduced the size of glycolytic fibres and tended to increase the proportion of oxidative fibres. It is likely that persistent *Atf5* overexpression maintains activation of the ISR, thereby reducing protein synthesis and/or increasing protein degradation and possibly apoptosis, resulting in inhibition of muscle growth, with overexpression of *Arg2* having a similar effect.

## Introduction

**Figure 1:**
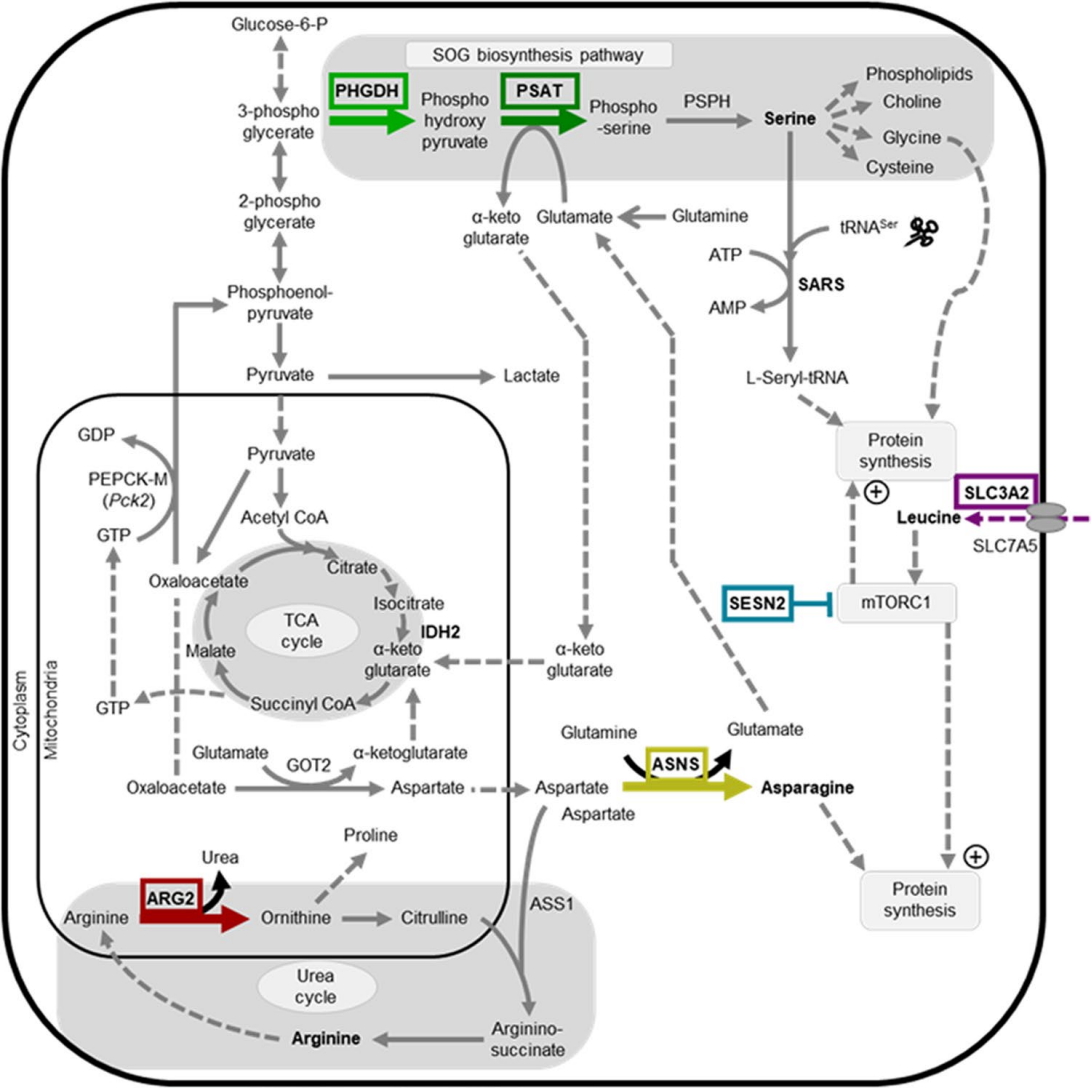
Schematic illustrating the role of AAV1-mediated targets associated with energy metabolism, protein synthesis and the urea cycle. Schematic highlights (in bold font with bold arrows) the different genes of interest (GOI) targeted for AAV1-mediated overexpression in this study and their function. GOI are involved in amino acid biosynthesis (Phosphoglycerate Dehydrogenase, PHGDH; Phosphoserine aminotransferase, PSAT1; Asparagine synthetase, ASNS), amino acid transport (Solute Carrier family 3 member 2, SLC3A2), the urea cycle (Arginase-2, ARG2) and an inhibitor of protein synthesis (Sestrin-2, SESN2). Relative mRNA expression was measured for GOIs, amino-acyl tRNA synthetase (Seryl-tRNA synthetase, SARS) and Isocitrate dehydrogenase-2 (IDH2) (highlighted in bold font only).

We previously^1,2^ identified a novel group of genes that were co-ordinately upregulated in pigs administered Ractopamine, a beta-adrenergic agonist (BA), and associated with muscle hypertrophy. Inferred Pathway Enrichment analysis highlighted that pathways associated with amino acid biosynthesis and transport were significantly upregulated in skeletal muscle of pigs following 3 days of BA administration; a large proportion of these genes related to tRNA charging, the urea cycle and serine biosynthesis^2^. These genes (summarised in Fig. 1) included, but was not limited to, Arginase-2 (ARG2) which catalyses the hydrolysis of arginine to ornithine and urea; Asparagine synthetase (ASNS) which catalyses the conversion of L-aspartate and L-glutamine to L-asparagine and L-glutamate; and Phosphoglycerate Dehydrogenase (PHGDH) and Phosphoserine aminotransferase (PSAT1), which catalyse the first and second steps of the serine biosynthesis pathway. Also upregulated were Sestrin-2 (SESN2), which negatively regulates mTOR signalling in response to leucine; and Solute Carrier family 3 member 2 (SLC3A2), which regulates intracellular calcium levels and transports L-type amino acids, such as leucine^1,2^. We subsequently determined that these targets were associated with an integrated stress response (ISR)^3^. It is unknown what role the proteins encoded by these genes play in regulating the growth of skeletal muscle, particularly in relationship to growth promoter stimulated muscle hypertrophy.

These targets are all reported to be transcriptionally upregulated by activating transcription factor-4 (ATF4), the main regulator of an ISR, by forming a heterodimer with CCAAT/enhancer binding proteins (C/EBP), particularly C/EBP-γ (encoded by CEBPG gene)^4–7^. There was no effect of BA treatment on ATF4 at the mRNA or protein level, however ATF5 and CEBPG mRNA were significantly upregulated at day 3 of BA treatment^2^. The function of ATF5 and C/EBP-γ in terminally differentiated muscle and their role in muscle growth is unknown, however this suggests that ATF5 and C/EBP-γ might be potential regulators of the muscle-specific BA-induced hypertrophic response observed in pigs.

We previously reported the effect of 28 days Adeno-associated virus (AAV) serotype 1-mediated overexpression of *Pck2* mRNA encoding the mitochondrial isoform of Phosphoenolpyruvate carboxykinase (PEPCK-M) in mouse *Tibialis anterior* (TA) muscle on muscle growth, utilising a contralateral limb design^8^. Importantly, AAV injection into the TA muscle resulted in equivalent infection of the adjacent *Extensor digitorum longus* (EDL)^8^, but much lower infection of the adjacent *Soleus* and *Gastrocnemius* muscles, meaning both TA and EDL muscles could be used for analyses. Here we utilise the same techniques to investigate whether the genes (*Arg2, Asns, Phgdh, Psat1, Sesn2, Slc3a2*) associated with the muscle-specific BA-induced hypertrophic response observed in pigs^1,2^, could stimulate muscle growth when overexpressed individually in mouse skeletal muscle. We also determined whether the ISR associated transcription factors, ATF5 and C/EBP-γ, could stimulate muscle growth when overexpressed individually or in combination. To our knowledge, this is the first time that these genes (*Arg2, Asns, Atf5, Cebpg Phgdh, Psat1, Sesn2, Slc3a2*) normally associated with the ISR have been investigated in terms of their potential role in muscle growth in vivo.

## Results

The contralateral limb design described previously^8^ was used to determine the effect of AAV1-mediated overexpression of each gene of interest (GOI). Previous work^8^ demonstrated that injection of an AAV-Pck2 construct into the TA muscle resulted in similar green fluorescence and overexpression of Pck2 in both the TA and EDL muscles. Hence, for all the studies described here, the EDL muscle was used for all RNA analyses and the TA for muscle histology.

### Effect of AAV-mediated overexpression of genes associated with an integrated stress response (ISR) on muscle growth

**Figure 2:**
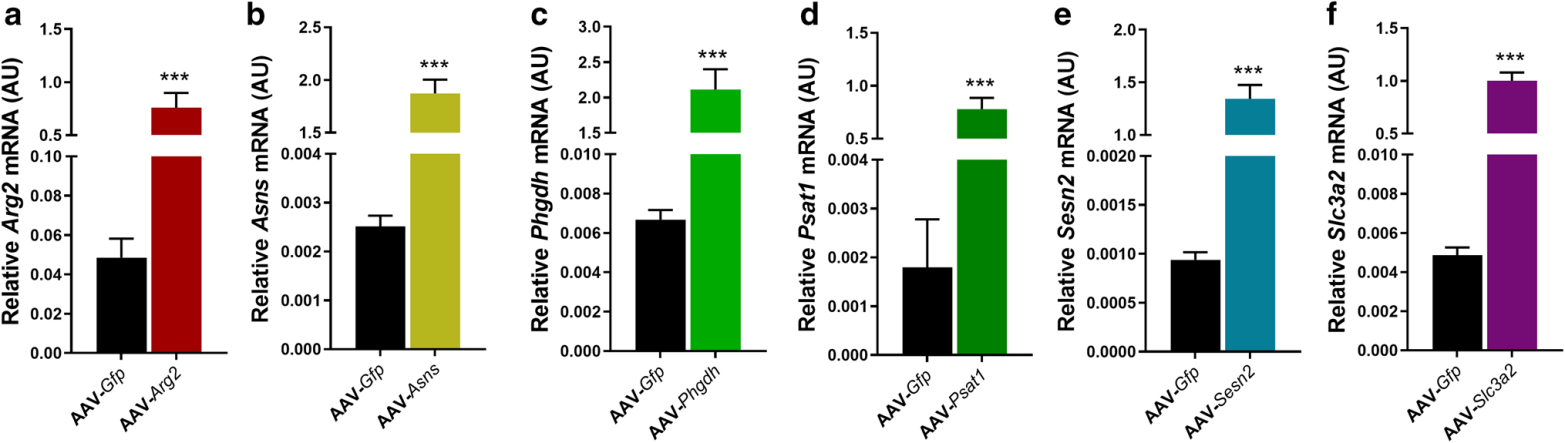
Validation of AAV-mediated overexpression of genes associated with an integrated stress response (ISR). Adeno-Associated Virus 1 (AAV)-mediated overexpression in the *Extensor Digitorum Longus* (EDL) muscle was validated by real-time quantitative PCR. Relative mRNA expression of target genes, normalised to OliGreen as a measure of total cDNA, are shown as follows: (**a**) Arginase-2 (*Arg2*), (**b**) Asparagine synthetase (*Asns*), (**c**) Phosphoglycerate Dehydrogenase (*Phgdh*), (**d**) Phosphoserine Aminotransferase-1 (*Psat1*), (**e**) Sestrin-2 (*Sesn2*) and (**f**) Solute Carrier family 3 member 2 (*Slc3a2*). Data presented as Means (n = 8, except AAV-*Sesn2* n = 10) ± SEM. ***(*P* < 0.001) indicates significantly different from contralateral control limb injected with AAV-*Gfp*.

At the mRNA level, Arginase-2 (AAV-*Arg2*), Asparagine synthetase (AAV-*Asns*), Phosphoglycerate Dehydrogenase (AAV-*Phgdh*), Phosphoserine Aminotransferase-1 (AAV-*Psat1*), Sestrin-2 (AAV-*Sesn2*) and Solute Carrier family 3 member 2 (AAV-*Slc3a2*) were all significantly increased in the EDL (*P* < 0.001; Fig. 2a–f).

**Figure 3:**
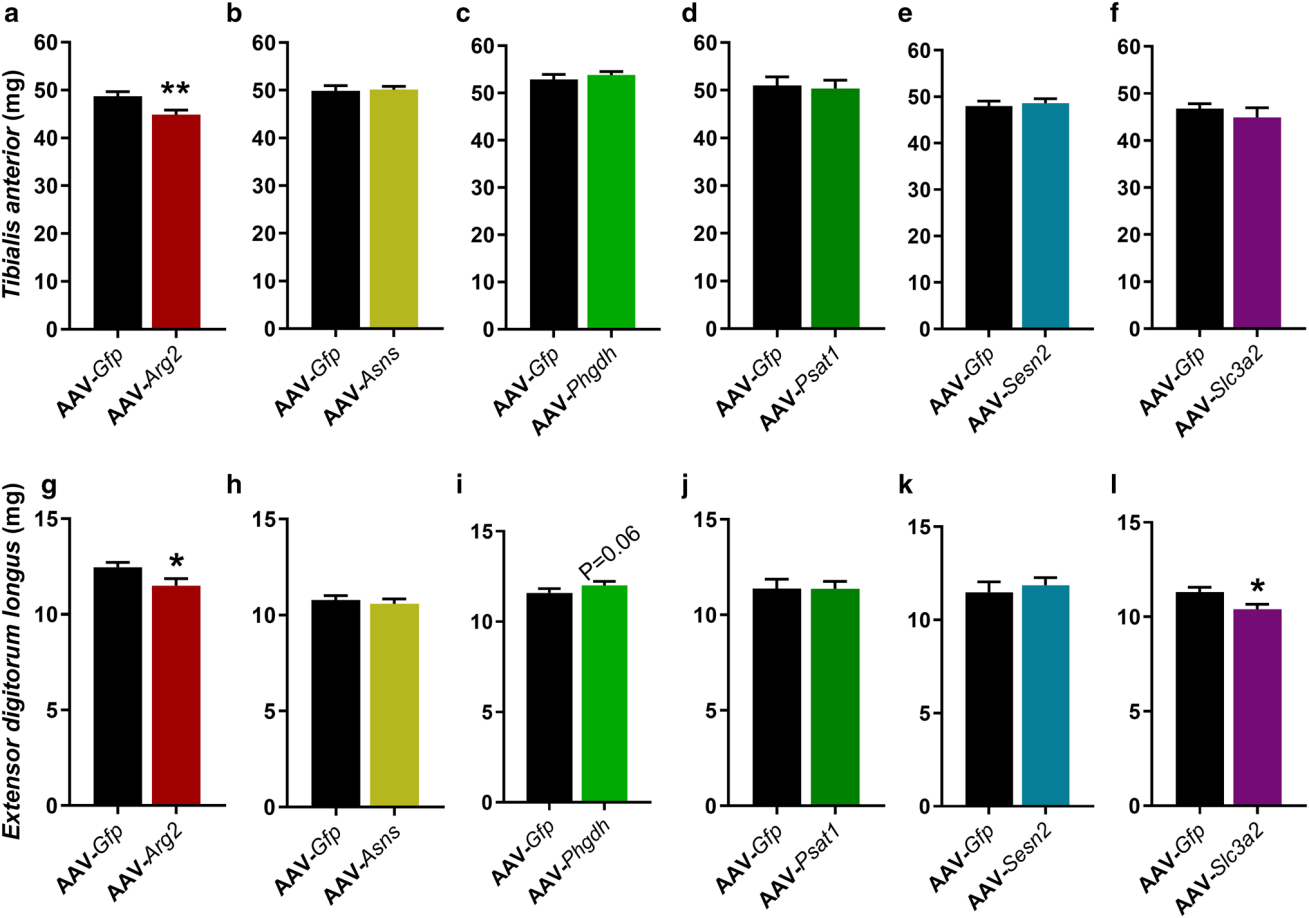
Effect of AAV-mediated overexpression of genes associated with an integrated stress response (ISR) on absolute muscle weights. Adeno-Associated Virus 1 (AAV) particles containing a gene of interest (GOI) were injected into mouse *Tibialis Anterior* (TA) muscle and a GFP control sequence alone (AAV-*Gfp*) into the contralateral TA, then maintained in their home cage for 28 days. GOI included Arginase-2 (AAV-*Arg2*), Asparagine synthetase (AAV-*Asns*), Phosphoglycerate Dehydrogenase (AAV-PHGDH), Phosphoserine Aminotransferase-1 (AAV-*Psat1*), Sestrin-2 (AAV-*Sesn2*) and Solute Carrier family 3 member 2 (AAV-*Slc3a2*). Data shown as Means (n = 8, except AAV-*Sesn2* n = 10) ± SEM for absolute TA (**a**–**f**) and *Extensor Digitorum Longus* (EDL; **g**–**l**) muscle weights. *(*P* < 0.05), **(*P* < 0.01) indicate significantly different from contralateral control limb injected with AAV-*Gfp*.

Then, we investigated if AAV-mediated overexpression of the individual genes for 28 days induced a change in muscle weights compared to their contralateral AAV-*Gfp* control limb. AAV-*Arg2* significantly reduced both TA (*P* = 0.005; Fig. 3a) and EDL (*P* = 0.01; Fig. 3g) absolute muscle weights, but AAV-*Asns*, AAV-*Phgdh*, AAV-*Psat1*, AAV-*Sesn2* and AAV-*Slc3a2* had no effect on TA muscle weights (Fig. 3b–f). Similarly AAV-*Asns*, AAV-*Psat1* and AAV-*Sesn2* had no effect on EDL muscle weights (Fig. 3h, j, k), whereas AAV-*Phgdh* tended to increase (*P* = 0.06; Fig. 3i) and AAV-*Slc3a2* significantly reduced EDL muscle weights (*P* = 0.04; Fig. 3l). There were no effects on *Gastrocnemius* or *Soleus* muscle weights (Supplementary Figure 1). We subsequently confirmed overexpression of the proteins for AAV-*Asns*, AAV-*Phgdh* and AAV-*Psat1* (Supplementary Figure 2), but we were unable to do so for the other genes as we had used up all the TA and EDL muscle samples.

### Effect of AAV-mediated overexpression on endogenous mRNA expression

**Figure 4:**
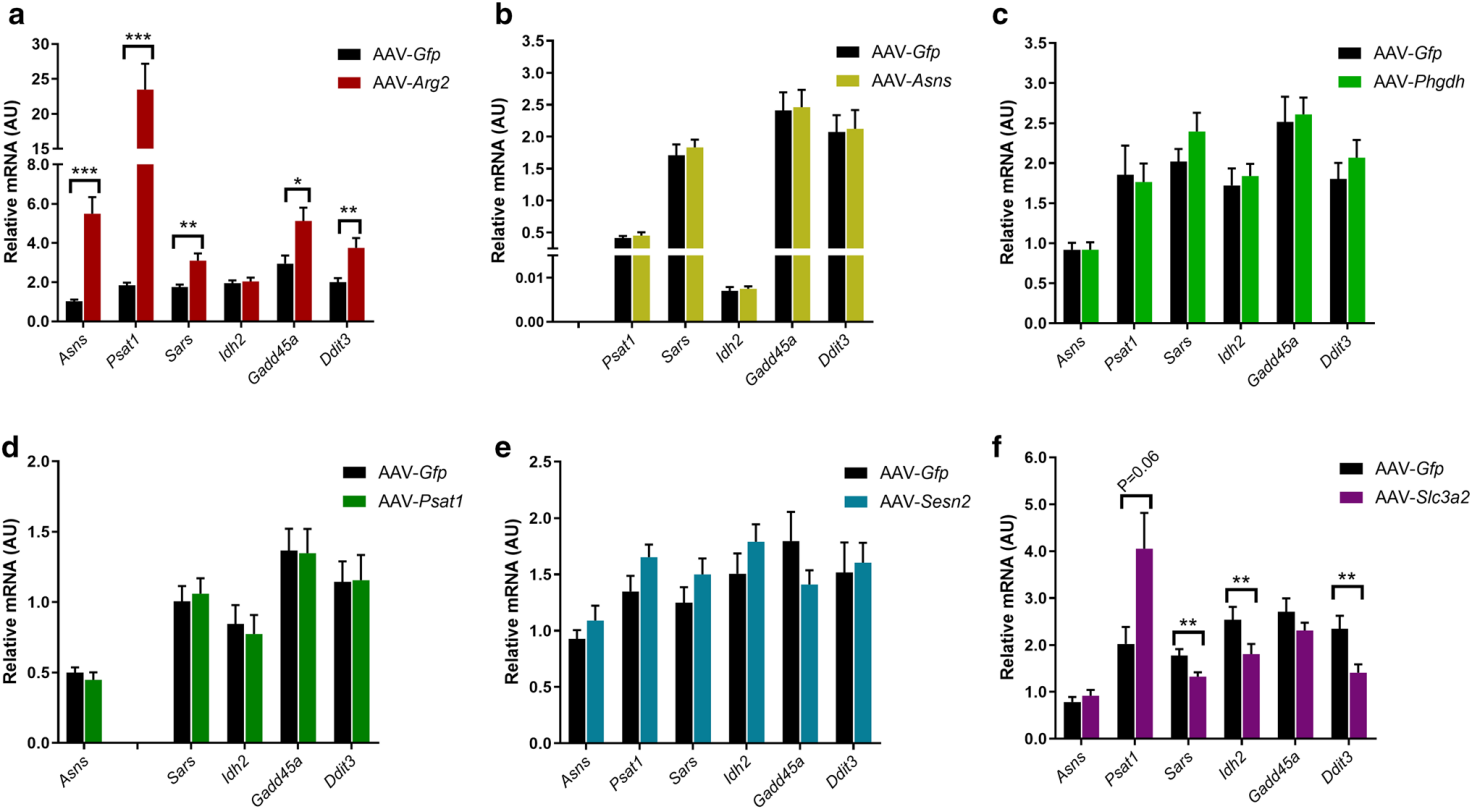
Effect of AAV-mediated overexpression of genes associated with an integrated stress response (ISR) on endogenous mRNA expression. Relative mRNA expression for Asparagine Synthetase (*Asns*), Phosphoserine Aminotransferase-1 (*Psat1*), Seryl-tRNA Synthetase (*Sars*), Isocitrate dehydrogenase-2 (*Idh2*), Growth Arrest And DNA-Damage-Inducible 45 Alpha (*Gadd45a*, also known as *Ddit1*) and DNA Damage Inducible Transcript 3 (*Ddit3*, also known as *CHOP*) were determined in the *Extensor Digitorum Longus* (EDL) muscle following 28 days AAV-mediated overexpression of (**a**) *Arg2*, (**b**) *Asns*, (**c**) *Phgdh*, (**d**) *Psat1*, (**e**) *Sesn2* and (**f**) *Slc3a2*. Relative *Asns* and *Psat1* mRNA expression were excluded from AAV-*Asns* and AAV-*Psat1* data respectively as these were reported in Fig. 2. All mRNA expression data were normalised to OliGreen, as a measure of total cDNA. Data presented as Means (n = 8, except AAV-*Sesn2* n = 10) ± SEM. *(*P* < 0.05) **(*P* < 0.01) and ***(*P* < 0.001) indicate significantly different to contralateral control limb injected with AAV-*Gfp*.

Next, we investigated if overexpression of the individual target genes altered mRNA expression of the other genes (*Asns*, *Psat1* and Seryl-tRNA Synthetase (*Sars*)) previously observed to be upregulated during BA induced pig skeletal muscle hypertrophy^1,2^. *Idh2* mRNA expression was also determined as an example of a gene that was downregulated in the pig studies. AAV-mediated overexpression of *Arg2* significantly increased *Asns*, *Psat1*, *Sars*, *Gadd45a* (also known as *Ddit1*) and *Ddit3* (also known as C/EBP Homologous Protein (CHOP)) mRNA (all P ≤ 0.01; Fig. 4a) but had no effect on *Idh2* mRNA. With the exception of their respective target genes (*Asns* and *Psat1*), AAV-mediated overexpression of Asns, *Phgdh, Psat1* and *Sesn2* had no effect on the expression of the other genes (Fig. 4b–e), whilst AAV-*Slc3a2* tended to increase *Psat1* (*P* = 0.06) and significantly reduced *Sars*, *Idh2* and *Ddit3* mRNA expression (all *P* < 0.01; Fig. 4f).

**Figure 5:**
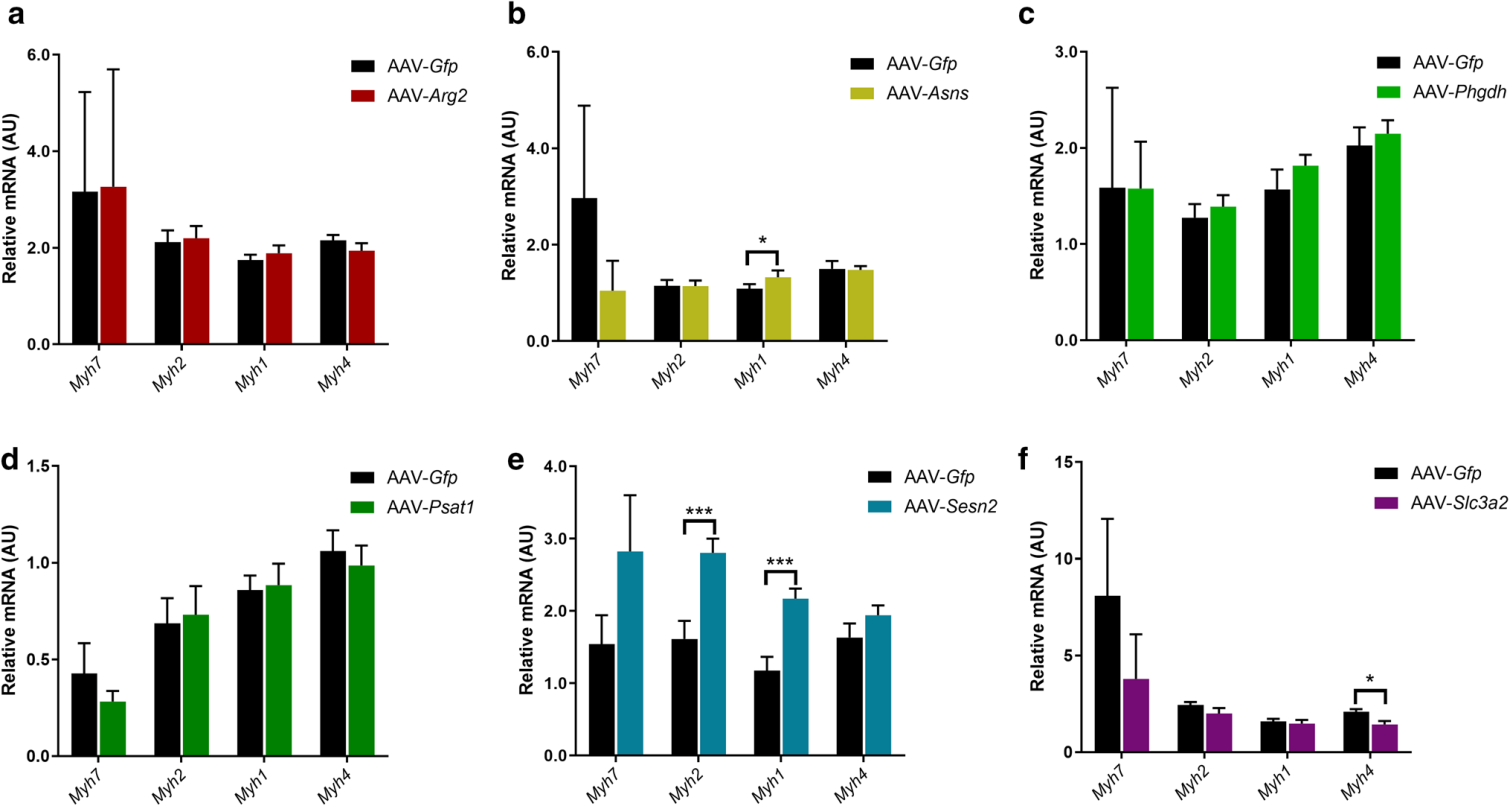
Effect of AAV-mediated overexpression of genes associated with an integrated stress response (ISR) on endogenous mRNA expression of myosin heavy chain (MyHC) isoforms. Relative mRNA expression for MyHC isoforms were determined in the *Extensor Digitorum Longus* (EDL) muscle following 28 days AAV-mediated overexpression of (**a**) *Arg2*, (**b**) *Asns*, (**c**) *Phgdh*, (**d**) *Psat1*, (**e**) *Sesn2* and (**f**) *Slc3a2*. *Myh7* encodes MyHC-I, *Myh2* encodes MyHC-IIA, *Myh1* encodes MyHC-IIX and *Myh4* encodes MyHC-IIB. All mRNA expression data were normalised to OliGreen, as a measure of total cDNA. Data presented as Means (n = 8, except AAV-*Sesn2* n = 10) ± SEM. *(*P* < 0.05) and ***(*P* < 0.001) indicate significantly different to contralateral control limb injected with AAV-*Gfp*.

Relative mRNA expression of the Myosin heavy chain (MyHC) isoforms were also measured as markers of muscle fibre type, to investigate whether overexpression of any of the genes might induce shifts in muscle fibre type. *Myh7* encodes slow oxidative MyHC-I, *Myh2* encodes fast oxidative MyHC-IIA, *Myh1* encodes fast oxidative-glycolytic MyHC-IIX and *Myh4* encodes fast glycolytic MyHC-IIB, with increased expression of the latter often associated with muscle hypertrophy. There were no effects of AAV-*Arg2*, AAV-*Phgdh* or AAV-*Psat* on the mRNA expression of any of the MyHC isoforms (Fig. 5a, c, d respectively). However, AAV-*Sesn2* significantly increased both *Myh2* and *Myh1* mRNA (both *P* < 0.001; Fig. 5e), AAV-*Asns* increased *Myh1* mRNA only (*P* = 0.05; Fig. 5b) and AAV-*Slc3a2* reduced *Myh4* mRNA (*P* = 0.02; Fig. 5f).

Hence, the AAV1-mediated overexpression of the individual genes were not sufficient to drive the BA-induced response observed in pigs^1,2^. Therefore, we investigated the effects of *Atf5* and/or *Cebpg* overexpression, as these are potential transcriptional regulators for the group of genes.

### AAV-mediated overexpression of activating transcription factor-5 (Atf5) ± Cebpg on muscle growth

As ATF5 forms a heterodimer with CCAAT/enhancer binding protein-γ (C/EBP-γ encoded by *Cebpg*), we utilised the contralateral limb design to study the effect of both AAV-*Atf5* and AAV-*Cebpg* being injected separately but also together (AAV-*Atf5* + *Cebpg*) on muscle growth.

**Figure 6:**
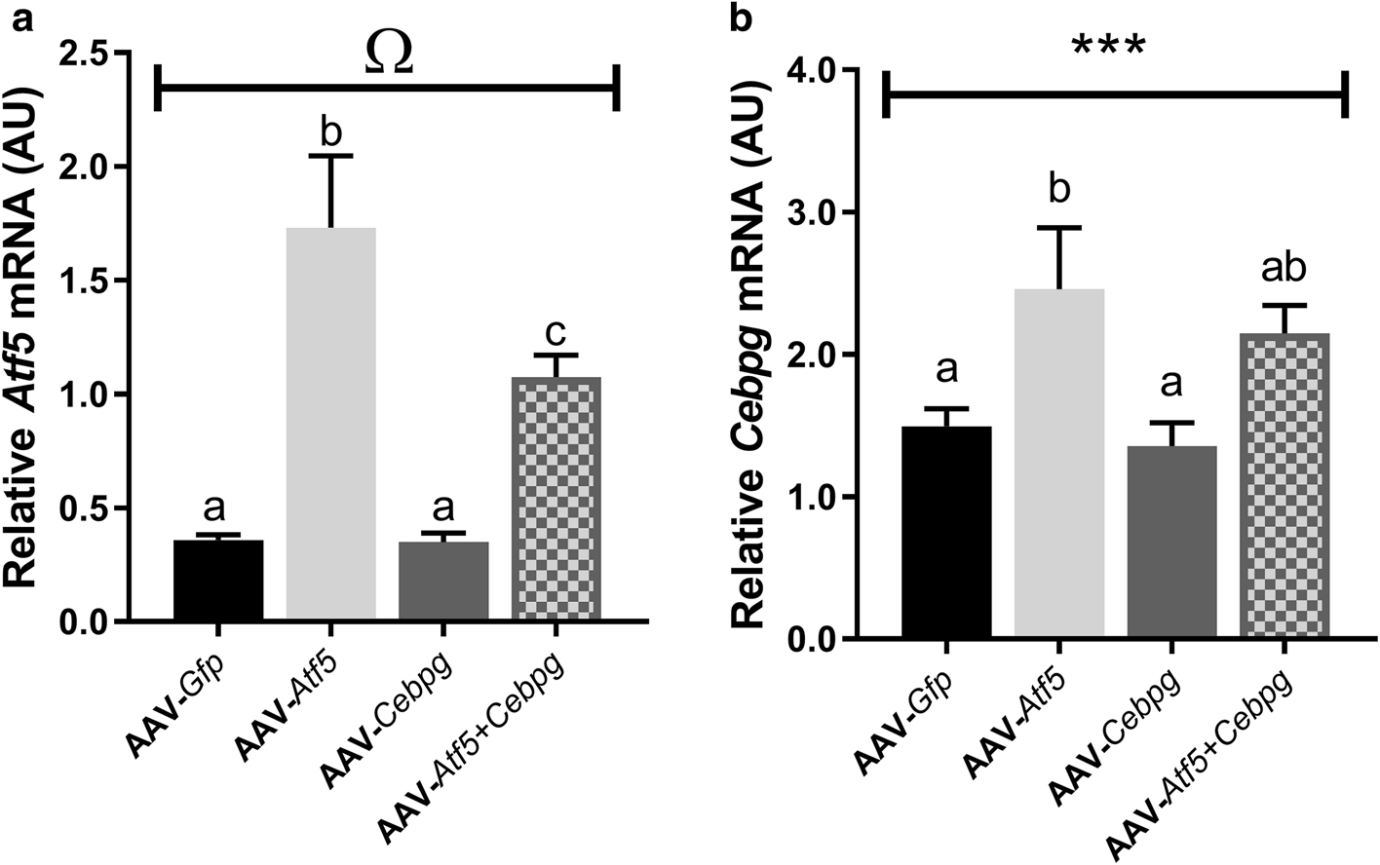
Validation of AAV-mediated overexpression of *Atf5* and *Cebpg* individually and in combination (AAV-*Atf5* ± *Cebpg*) at the mRNA level. Relative mRNA expression of (**a**) Activating transcription factor-5 (*Atf5*) and (**b**) CCAAT/enhancer binding protein gamma (*Cebpg*) were determined in the *Extensor Digitorum Longus* (EDL) muscle following 28 days AAV-mediated overexpression. mRNA expression data was normalised to OliGreen, as a measure of total cDNA. Data presented as Means (n = 8, except n = 24 for AAV-*Gfp*) ± SEM. Ω indicates a trend (*P* = 0.08) for an AAV-*Atf5* × AAV-*Cebpg* interaction. Different letters indicate significant differences (*P* < 0.05). ***P < 0.001 indicates significant effect of AAV-*Atf5* (with or without *Cebpg*).

QPCR was performed to confirm whether *Atf5* and *Cebpg* were overexpressed at the mRNA level 28 days after AAV injections. There tended to be an AAV-*Atf5* × AAV-*Cebpg* interaction (*P* = 0.08; Fig. 6a) for relative *Atf5* mRNA expression, but not *Cebpg* mRNA (Fig. 6b). There was a significant increase in relative *Atf5* mRNA following AAV-mediated overexpression of *Atf5* for 28 days (both AAV-*Atf5* and AAV-*Atf5* + *Cebpg P* < 0.001; Fig. 6a), but not AAV-*Cebpg*. There was also a significant increase in *Cebpg* mRNA following AAV-mediated *Atf5* overexpression (*P* = 0.001; Fig. 6b), but not AAV-*Cebpg*. This demonstrates that transduction with AAV-*Atf5* alone successfully upregulated both *Atf5* and *Cebpg* at the mRNA level, but there were no effects of AAV-*Cebpg* alone. This may mean either that the AAV-*Cebpg* construct was not functional or that the *Cebpg* gene expression is tightly regulated, but other data suggests that the construct was functional.

**Figure 7:**
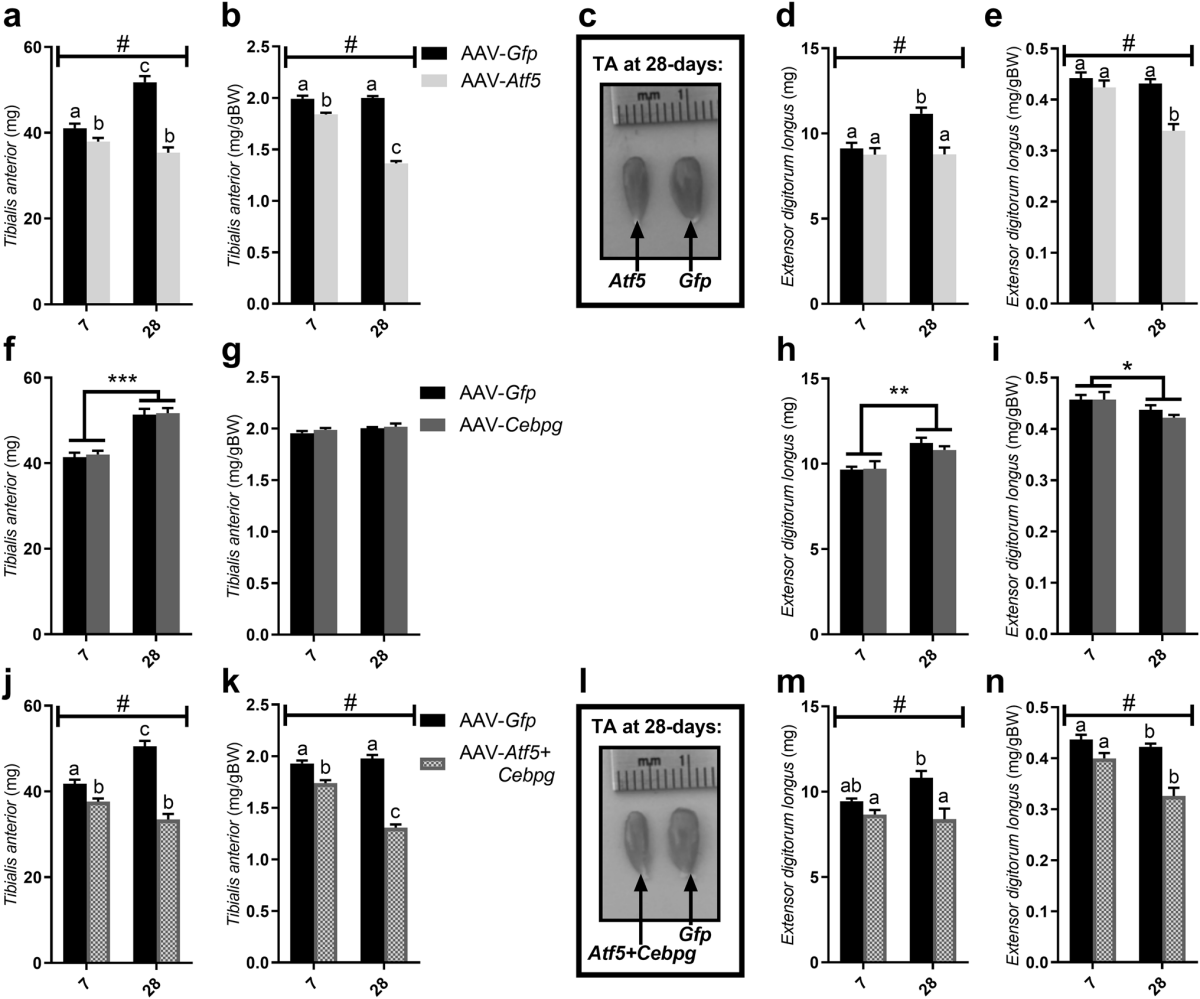
Effect of 7 and 28 days of AAV-mediated overexpression of *Atf5* and *Cebpg* individually and in combination (AAV-*Atf5* ± *Cebpg*) on mouse muscle weights. Absolute and muscle weights relative to body weight (BW) for *Tibialis anterior* (TA; **a**/**b**, **f**/**g**, **j**/**k**) and *Extensor digitorum longus* (EDL; **d**/**e**, **h**/**i**, **m**/**n**) following 7 and 28 days of AAV-mediated overexpression of *Atf5* (**a**–**e**), *Cepbg* (**f**–**i**) or both (AAV-*Atf5* + *Cebpg*, **j**–**n**). Representative photographs of TA muscle transduced with (**c**) AAV-*Atf5* or (**l**) AAV-*Atf5* + *Cebpg* and the contralateral control TA from the same animal transduced with AAV-*Gfp* for 28-days. Data presented as Means (n = 8) ± SEM. # indicates a significant AAV × time interaction (*P* < 0.01). Different letters indicate significant differences (*P* < 0.05). *(*P* < 0.05), **(*P* < 0.01) and ***(*P* < 0.001) indicate significant differences in muscle weights at day 28 compared to day 7.

There were significant AAV × time interactions following AAV-mediated overexpression of *Atf5* alone and AAV-*Atf5* + *Cebpg* on both absolute and relative muscle weights for TA (all *P* < 0.001, Fig. 7a, b and 7j, k respectively) and EDL (*P* < 0.001, Fig. 7d, e and *P* < 0.01, Fig. 7m, n). Absolute muscle weights for both TA and EDL increased with time (day 28 compared to day 7), whilst muscle weights relative to body weight remained constant. There were significant reductions in absolute TA muscle weights 7 days after injection with AAV-*Atf5* alone and AAV-*Atf5* + *Cebpg* compared to their contralateral control limb TA. Both TA and EDL muscle weights were reduced further after 28 days of transduction as is clearly observed in Fig. 7c, l respectively. Indeed, both AAV-*Atf5* and AAV-*Atf5* + *Cebpg* appeared to blunt the growth of both TA and EDL muscles, resulting in a dramatic reduction in muscle weights relative to body weight 28 days after AAV injections.

There was no effect of AAV-*Cebpg* on absolute or relative muscle weights and no AAV × time interaction. There were significant increases in absolute TA (*P* < 0.001; Fig. 7f) and EDL (*P* = 0.004; Fig. 7h) muscle weights, as well as relative EDL weights (*P* = 0.02; Fig. 7i) at 28 days compared to 7 days, but no effect of time for relative TA muscle weights (Fig. 7g).

### Effect of AAV-Atf5 ± Cebpg on TA muscle composition

Succinate Dehydrogenase (SDH) staining is an indicator of muscle fibres oxidative capacity and mainly identifies type-I and -IIA fibres by staining them a darker blue compared to type-IIX and -IIB muscle fibres that are predominantly dependent on glycolytic metabolism and therefore remain unstained.

**Figure 8:**
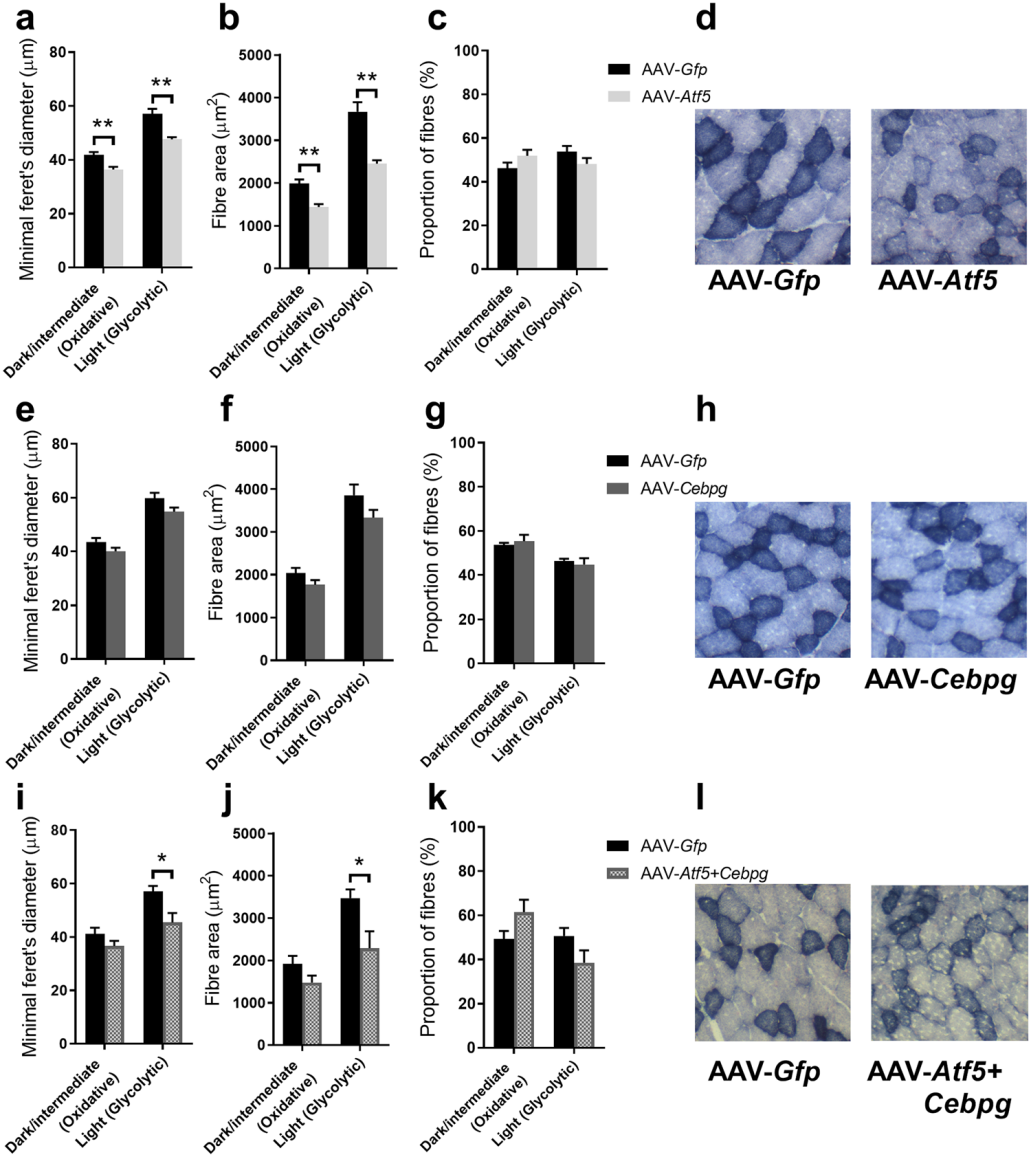
Effect of AAV-mediated overexpression of *Atf5* and *Cebpg* individually and in combination (AAV-*Atf5* ± *Cebpg*) on muscle fibre size and composition of transduced TA. Succinate Dehydrogenase (SDH) staining was performed on 10 µm sections collected from TA muscle transduced with AAV-*Atf5* (**a**–**d**), AAV-*Cebpg* (**e**–**h**) or AAV-*Atf5* + *Cebpg* (**i**–**l**) for 28 days and the contralateral TA muscle transduced with AAV-*Gfp*. Average minimal feret’s diameters (**a**, **e**, **i**) and fibre areas (**b**, **f**, **j**) were determined for 15 oxidative (dark/intermediate) and 15 glycolytic fibres per field of view. The proportion of dark/intermediate (oxidative) and light (glycolytic) fibres (**c**, **g**, **k**) were determined as a percentage of the total number of fibres per field of view. Data presented as Means (n = 4–6) ± SEM, where the average of two or three fields of view were taken per sample). Unpaired *t*-tests were performed, *(*P* < 0.05) and **(*P* < 0.01) indicate a significant difference between AAV-GOI compared to AAV-*Gfp* control. Representative images were captured at 10 × magnification (**d**, **h**, **l**).

AAV-*Atf5* overexpression for 28 days resulted in a significant reduction of minimal feret’s diameter and fibre area for both oxidative (dark/intermediate SDH stain) and glycolytic (light) fibres (all *P* < 0.01; Fig. 8a, b). However, there was no effect on the proportion of oxidative to glycolytic fibres compared to the contralateral control leg muscle (Fig. 8c). This is clearly supported by the representative images in Fig. 8d that show AAV-*Atf5* overexpression resulted in smaller muscle fibres compared to the AAV-*Gfp* control. The combined AAV-*Atf5* + *Cebpg* also resulted in a significant reduction of minimal feret’s diameter and fibre area, but only for glycolytic fibres (both *P* < 0.05; Fig. 8i, j). There was also a tendency for a decrease in the proportion of glycolytic fibres and an increase in oxidative fibres (*P* = 0.09; Fig. 8k). In contrast, AAV-*Cebpg* had no effect on minimal feret’s diameter or fibre area of oxidative or glycolytic muscle fibres (Fig. 8e, f). There was also no effect on the proportion of oxidative and glycolytic fibres compared to the contralateral control leg muscle (Fig. 8g). Representative images for AAV-*Atf5*, AAV-*Cebpg* and AAV-*Atf5* + *Cebpg* compared to the AAV-*Gfp* control are shown in Fig. 8d, h l respectively.

### Effect of AAV-Atf5 ± Cebpg on endogenous mRNA expression

**Figure 9:**
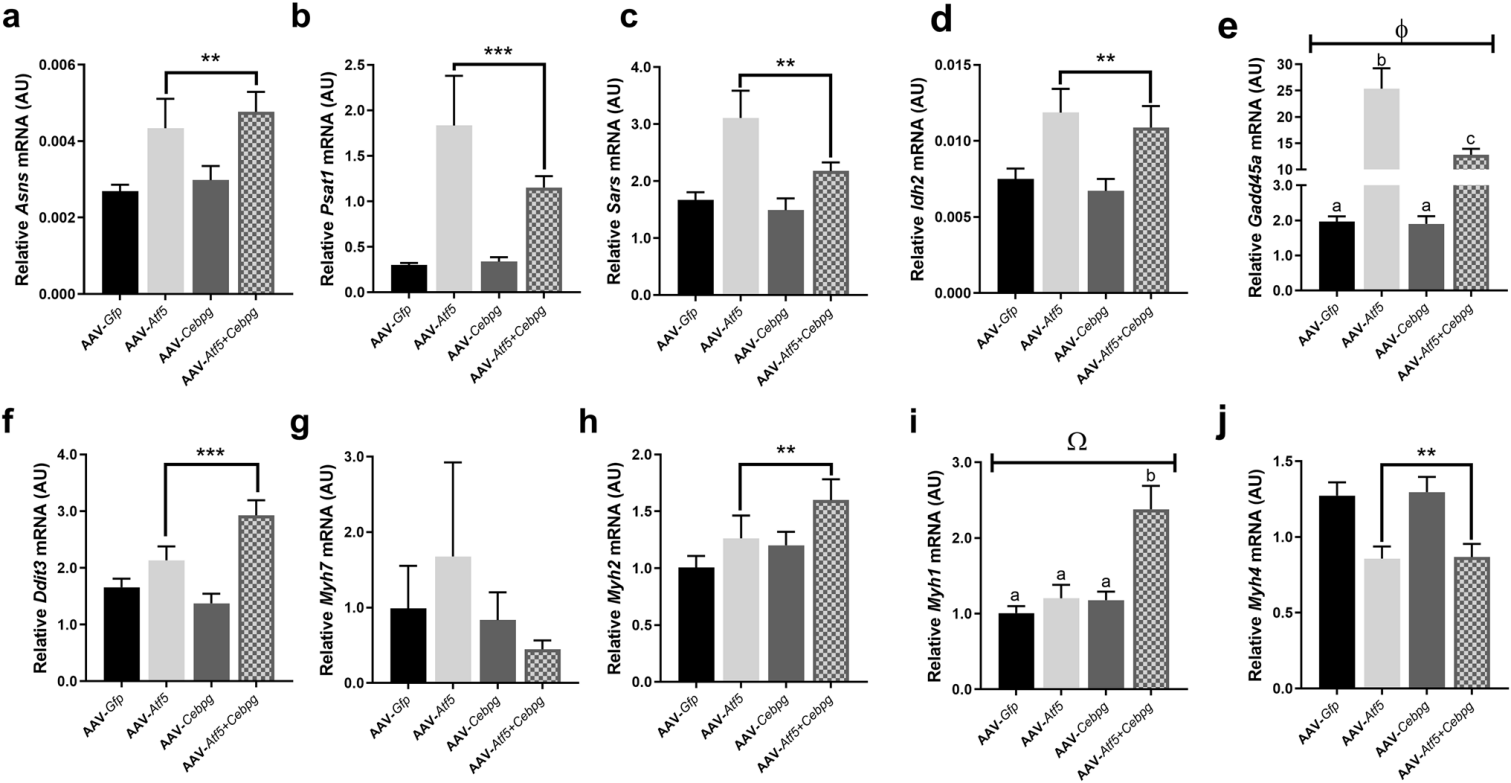
Effect of AAV-mediated overexpression of *Atf5* and *Cebpg* individually and in combination (AAV-*Atf5* ± *Cebpg*) on endogenous mRNA expression. Relative mRNA expression for (**a**) Asparagine synthetase (*Asns*), (**b**) Phosphoserine Aminotransferase-1 (*Psat1*), (**c**) Seryl-tRNA Synthetase (*Sars*), (**d**) Isocitrate dehydrogenase-2 (*Idh2*), (**e**) Growth Arrest and DNA-Damage-Inducible 45 Alpha (*Gadd45a*, also known as *Ddit1*), (**f**) DNA Damage Inducible Transcript 3 (*Ddit3*, also known as *CHOP*), and (**g**–**j**) Myosin heavy chain (MyHC) isoforms were determined in the *Extensor Digitorum Longus* (EDL) muscle following 28 days AAV-mediated overexpression. (**g**) *Myh7* encodes slow oxidative MyHC-I, (**h**) *Myh2* encodes fast oxidative MyHC-IIA, (**i**) *Myh1* encodes fast oxidative-glycolytic MyHC-IIX and (**j**) *Myh4* encodes fast glycolytic MyHC-IIB. All mRNA expression data was normalised to OliGreen, as a measure of total cDNA. Data presented as Means (n = 8, except n = 24 for AAV-*Gfp*) ± SEM. φ indicates a significant AAV-*Atf5* × AAV-*Cebpg* interaction (*P* = 0.005), and Ω indicates a trend for an interaction (*P* = 0.06); different letters indicate statistical significance (*P* < 0.05). **P < 0.01, ***P < 0.001 indicates significant effect of AAV-*Atf5*.

Since ATF5 and C/EBP-γ are thought to be transcriptional regulators for the ISR, the expression of *Asns*, *Psat1, Sars, Gadd45a* and *Ddit3* (all ISR genes), as well as *Idh2* mRNA were again determined. There was a significant AAV-*Atf5* × AAV-*Cebpg* interaction (*P* = 0.005; Fig. 9e) for relative mRNA expression of *Gadd45a*, but not the other ISR genes. This was due to AAV-*Atf5* increasing *Gadd45a* mRNA expression, but this was reduced (but still higher than control) when in combination with AAV-*Cebpg*, whereas AAV-*Cebpg* alone had no effect. AAV-*Atf5* significantly increased *Asns*, *Psat1*, *Sars*, *Idh2* and *Ddit3* mRNA expression (all *P* < 0.01; Fig. 9a–d, f respectively), whereas AAV-*Cebpg* had no effect on any of these genes. This indicates that AAV-mediated *Atf5* overexpression alone was capable of upregulating the mRNA expression for all these downstream target genes involved in an ISR.

There was no effect of AAV-*Atf5* or AAV-*Cebpg* mediated overexpression on *Myh7* mRNA (Fig. 9g). However, there was a significant increase in *Myh2* (*P* = 0.006; Fig. 9h) and a reduction in *Myh4* (*P* = 0.002; Fig. 9j) mRNA expression following AAV-*Atf5* overexpression, but not AAV-*Cebpg*. There tended to be an AAV-*Atf5* × AAV-*Cebpg* interaction for *Myh1* mRNA (*P* = 0.06; Fig. 9i), due to a significant increase with the combination of AAV-*Atf5* + *Cebpg*, but no effects of AAV-*Atf5* or AAV-*Cebpg* separately. This appears to indicate that AAV-*Cebpg* is contributing to this response, indicating the AAV-*Cebpg* construct was functional, therefore suggesting that the lack of change in *Cebpg* mRNA (Fig. 6b) was due to *Cepbg* gene expression being tightly regulated. Unfortunately we were unable to investigate whether there were effects on protein levels for Atf5 and/or Cebpg, as we had used up the TA and EDL muscles in the molecular and histological analyses.

## Discussion

The aim of this study was to investigate the effects of AAV1-mediated overexpression of a group of genes in mouse skeletal muscle. This group of genes were upregulated in BA treated porcine skeletal muscle^1,2^, which included downstream targets (*Arg2*, *Asns*, *Phgdh*, *Psat1*, *Sesn2*, *Slc3a2*) and regulators (*Atf5*, *Cebpg*) of an ISR. To our knowledge, this is the first time these genes have been selectively overexpressed in mouse muscle to determine their effects on muscle growth.

We utilised a method described previously^8^ to provide localised overexpression (restricted to the hindlimb muscles) and compared the effect of overexpression in one leg compared to the contralateral leg injected with an AAV-*Gfp* control. Other groups have used similar approaches in mice and demonstrated that AAV-mediated overexpression of Follistatin resulted in muscle hypertrophy, as TA muscle weight was significantly increased compared to the contralateral control TA^9^. We successfully overexpressed the level of individual genes (*Arg2*, *Asns*, *Phgdh*, *Psat1*, *Sesn2*, *Slc3a2*) at the mRNA level following AAV1-mediated transduction for 28 days, confirming overexpression at the protein level for *Asns*, *Phgdh* and *Psat1*. Contrary to our original hypothesis that overexpression might induce hypertrophy, overexpression of the individual downstream ISR targets had no effect (AAV-*Asns*, -*Phgdh*, -*Psat1*, -*Sesn2*, -*Slc3a2*) or resulted in muscle atrophy (AAV-*Arg2*) of the transduced TA muscle. Arginase has two isoforms: cytosolic ARG1 and mitochondrial ARG2, which catalyse the conversion of arginine to ornithine and urea as part of the urea cycle. ARG1 is mainly found in the liver whilst ARG2 is ubiquitously expressed in various tissues^10^ and is highly expressed in certain prostate^11^ and pancreatic cancers^12,13^, although lower expression compared to non-malignant tissue has also been reported^14–16^. ARG2, but not ARG1, was significantly increased following BA-induced muscle hypertrophy in pigs^2^, whereas here AAV-*Arg2* overexpression resulted in a significant reduction in mouse TA and EDL muscle weights. Relative mRNA expression of *Asns*, *Psat1*, *Sars*, *Gadd45a* and *Ddit3* were all significantly increased in the EDL; which might indicate the activation of an ISR, possibly due to increased arginine hydrolysis, resulting in nutrient deprivation. This potentially demonstrates that by selectively overexpressing *Arg2* alone, a shift in protein synthesis is induced through the upregulation of other enzymes involved in amino acid synthesis (e.g. ASNS and PSAT1 for asparagine and serine biosynthesis respectively) and aminoacyl-tRNA-synthetases in protein translation (e.g. SARS). However, since *Gadd45a* and *Ddit3* are known to be activated by endoplasmic reticulum (ER) stress and to subsequently promote apoptosis, the increases in their expression may be partly responsible for the muscle atrophy^17–19^.

We reported previously that PEPCK-M overexpression also resulted in slight muscle atrophy^8^. PEPCK-M is reported to be upregulated in certain forms of cancer leading to reprogramming of energy metabolism to aid tumour cell growth and progression^20,21^. Similarly, ASNS, PHGDH, PSAT1 and SLC3A2 are all highly expressed in different forms of cancer^22,23^, however little is known about their role in skeletal muscle growth and hypertrophy. There was no effect on TA muscle weight following AAV-*Slc3a2* overexpression, whereas EDL muscle weight was significantly reduced. The lack of response may be a result of increased variability in the larger TA (~ 45 mg) versus smaller EDL (~ 10 mg) muscle or slight differences in fibre-type composition between the different hindlimb muscles; however this would need further investigation with increased replicates to clarify. In contrast, SESN2 expression is reported to be largely suppressed in many cancers, despite the high level of reactive oxygen species, but there are also reports of increased SESN2 expression in some cancers^24^. SESN2 is an inhibitor of mTOR and therefore protein synthesis, so it was surprising that AAV-*Sesn2* had no effect on muscle weight, particularly as there were significant increases in both *Myh2* and *Myh1* mRNA expression (encode MyHC-IIA and –IIX respectively). It is possible that AAV-*Sesn2* overexpression (or other AAV targets) for longer than 28 days might have induced a muscle phenotype, however we show here and previously^8^ that 28 days is adequate to detect changes in TA and EDL muscle weights.

The BA-induced hypertrophic response observed in porcine skeletal muscle was associated with a clear coordinated upregulation of a group of genes^1,2^. Although AAV-*Arg2* overexpression was capable of inducing a selection of these genes at the mRNA level (*Asns*, *Psat1, Sars*) it was also associated with muscle atrophy and the upregulation of *Gadd45a* and *Ddit3* mRNA, both of which are associated with ER stress and apoptosis^17–19^. We hypothesised that it might be necessary to overexpress a regulator for this group of genes in order to recreate the synchronous coordinated response and therefore induce muscle hypertrophy. Using HepG2 cells, ATF5 overexpression has been reported to transactivate ASNS, a marker of ER stress^25^, and co-transfection with *Cebpg* induced *Pgc-1α* promoter activity^26^. *Atf5* and *Cebpg* demonstrated the same response profile as the group of target genes^2^ and therefore offer plausible candidates that might regulate this hypertrophic response. However, we demonstrate for the first time that chronic overexpression of *Atf5* (alone or combined with *Cebpg*) for 28 days in mouse TA muscle reduced TA muscle weights, as well as average fibre diameter and fibre area. Relative isocitrate dehydrogenase-2 (*Idh2*) mRNA expression was significantly upregulated following AAV-*Atf5* overexpression, therefore we also report the novel observation of ATF5 as a potential transcriptional regulator of *Idh2*. *Idh2*, a marker of oxidative metabolism, was downregulated in porcine skeletal muscle treated with BA^1^, therefore supporting the observed fibre-type shift to a more fast-twitch, glycolytic phenotype indicated by an increase in *Myh4* (encodes MyHC-IIB) mRNA, as well as a decrease in *Myh1* (MyHC-IIX) and *Myh2* (MyHC-IIA)^1^. The reverse was observed in this study, where chronic overexpression of either *Atf5* alone or *Atf5* + *Cebpg* blocked the normal muscle growth and maturation. AAV-*Atf5* reduced muscle fibre size (diameter and area) of oxidative and glycolytic fibres, whereas AAV-*Atf5* + *Cebpg* only reduced the size of glycolytic fibres. This inhibition of muscle growth was associated with a significant increase in *Myh2* and *Idh2* mRNA and a decrease in *Myh4* mRNA following AAV-mediated overexpression of *Atf5* alone and in combination with *Cebpg* (AAV-*Atf5* + *Cebpg*), therefore indicating a shift in oxidative capacity. AAV-*Atf5* had no effect on the proportion of oxidative versus glycolytic fibres, however AAV-*Atf5* + *Cebpg* tended to increase the proportion of oxidative fibres in the transduced TA muscle. AAV-*Atf5* + *Cebpg* appeared to have a different phenotypic effect compared to AAV-*Atf5* alone, therefore suggesting that AAV-*Cebpg* was functional, despite no changes in *Cebpg* mRNA expression and AAV-*Cebpg* alone having no effect. It would be useful to confirm overexpression of both *Atf5* and *Cebpg* at the protein level, particularly at an earlier time point, but we were unable to do so due to the EDL and TA muscles being used up in the molecular (gene expression) and histology (muscle fibre type and size) analyses respectively. The role of ATF5 in muscle growth is unknown, however this highlights that ATF5 might be the key regulator for this response. It is possible that ATF5 upregulation mimics a state of nutrient deprivation, therefore inducing an integrated stress or similar stress response. Under normal conditions, once nutrient levels have returned to normal, the ISR would be terminated and therefore *Atf5* downregulated. In our study, *Atf5* levels were maintained at a high level for 28 days leading to chronic activation of the ISR, as indicated by increased *Asns, Psat1, Sars, Gadd45a* and *Ddit3* mRNA, and as a result potentially suppresses protein synthesis or increases protein degradation and apoptotic processes, therefore leading to inhibition of muscle growth. As BA treatment was administered to pigs daily^1,2^, this possibly resulted in a pulsatile activation of the ISR. A similar induction of *Atf5* overexpression might be necessary in order to induce muscle hypertrophy, therefore future work should investigate the administration of inducible short-term pulses of *Atf5* overexpression in mouse muscle rather than the chronic overexpression studied here. It should be noted that the increases in mRNA expression observed here in AAV infected mice were substantially greater than those observed previously in BA treated pigs, with the fold changes observed in mice being around 10–80 times more than in pigs, with a range between 1.8-fold (for *Arg2*) and 150 fold (for *Sens2*) greater increases in AAV mice. Hence, better control of both the level of overexpression and the time of overexpression (acute vs chronic) may be necessary. Alternatively, it could simply be that pigs and mice respond differently and therefore mice are not a good model for pigs.

## Conclusion

We clearly demonstrate for the first time that AAV-mediated overexpression of either *Arg2* or *Atf5* in mouse TA muscle result in muscle atrophy associated with a co-ordinated induction of the mRNA expression of ISR genes (*Asns*, *Psat1*, *Sars, Gadd45a* and *Ddit3*) in skeletal muscle. We also report that AAV-*Atf5* increased *Idh2* mRNA expression in muscle, indicating that ATF5 may be a transcription factor that binds to the *Idh2* gene promoter. We hypothesise that the chronic overexpression of AAV-*Atf5* induces muscle atrophy because it prevents termination of the ISR (or similar stress response), leading to the induction of markers of ER stress and apoptosis (*Gadd45a* and *Ddit3*). Future work should investigate the effects of short-term inducible *Atf5* overexpression for possible induction of muscle hypertrophy.

## Methods

### Animals

Male C57BL6/J mice (aged 5–6-weeks; Charles River UK Ltd.) were kept at a controlled temperature (21 ± 1 °C) and held on 12 h light: 12 h dark photoperiod (lights off at 19:00) with ad libitum access to rodent chow (Teklad 2018, Envigo, UK) and filtered water. Mice were group housed (n = 2–4 per cage) and health status monitored throughout. All animal procedures were approved by The University of Nottingham Ethical Review Committee and conducted in accordance with the UK Animals (Scientific Procedures) Act of 1986 (Project License PPL PFBB3B51F), meaning that they are in compliance with ARRIVE guidelines.

### Adeno-associated virus (AAV)

The following vectors were designed using the VectorBuilder platform (https://en.vectorbuilder.com/; previously Cyagen Biosciences, now VectorBuilder Inc) and the specified mouse mRNA sequence accession numbers: pAAV-CMV-*Arg2*-T2A- *EmGfp* (abbreviated to AAV-*Arg2*; Accession number NM_009705.3), pAAV-CMV-*Asns*-T2A- *EmGfp* (AAV-*Asns*; XM_006505093.2), pAAV-CMV-*Atf5*-T2A- *EmGfp* (AAV-*Atf5*; NM_030693.2), pAAV-CMV-*Cebpg*-T2A- *EmGfp* (AAV-*Cebpg*; XM_006539500.2), pAAV-CMV-*Phgdh*-T2A- *EmGfp* (AAV-PHGDH; NM_016966.3), pAAV-CMV-*Psat1*-T2A- *EmGfp* (AAV-*Psat1*; NM_177420.2), pAAV-CMV-*Sesn2*-T2A- *EmGfp* (AAV-*Sesn2*; NM_144907.1), pAAV-CMV-*Slc3a2*-T2A- *EmGfp* (AAV-*Slc3a2*; NM_001161413.1) and pAAV-CMV-*EmGfp* (AAV-*Gfp*). The stop codon for each gene of interest (GOI) was removed and a viral T2A sequence included to ensure co-translational cleavage of the target protein from the green fluorescent protein (GFP)^27^. Hence both proteins are expected to be expressed in equimolar concentrations and the GFP acts as a marker of target protein expression^28^.

### Intramuscular AAV injections

As previously described^8^, mice were administered with single 20 µl injections containing approximately 1.2 × 10^10^ viral genome copies (GC) into both the left and right *Tibialis anterior* (TA) muscles while under recoverable anaesthesia (1.5% Isoflurane). We again used a contralateral leg design such that each AAV-GOI (target gene linked with GFP tag) was injected into the left TA and equivalent number of AAV1 particles containing GFP (AAV-*Gfp*) injected into the right TA as the control. Mice were then recovered from anaesthesia and maintained in their home cages for 28 days.

Single and combined injections were performed for AAV-*Atf5* and AAV-*Cebpg*, such that mice were injected with 3.1 × 10^9^ viral GC (in 20 µl) of AAV-*Atf5*, AAV-*Cebpg* or AAV-*Atf5* + AAV-*Cebpg* (3.1 × 10^9^ GC of each AAV in a total volume of 20 µl) into the left TA and equivalent GC number of AAV-*Gfp* into the right TA. Mice were then maintained in their home cages for 7 or 28 days.

### Termination

Mice were euthanized by cervical dislocation and the TA, *Extensor digitorum longus* (EDL), *Soleus* and *Gastrocnemius* hindlimb muscles dissected, weighed and snap frozen on dry-ice (n = 8 animals per GOI, except AAV-*Sesn2* n = 10). For mice injected with AAV-*Atf5* and/or AAV-*Cebpg*, TA and EDL muscles were collected 7 and 28 days after AAV injections (n = 8 animals per AAV per time-point). TA muscles were either transversely cut (AAV-*Asns*, -*Phgdh*, -*Psat*) and the distal half embedded with OCT or whole muscles were embedded in OCT (all other AAV targets), then OCT embedded muscles were frozen using dry ice cooled isopentane for muscle sectioning. The proximal half of the TA and whole EDL muscles were snap frozen on dry ice. All samples were stored at − 80 °C prior to analyses.

### Gene expression analyses

Gene expression was determined on whole EDL. Total RNA was extracted and quantitative RT-PCR carried out using the SYBR green method as previously described^29^. Relative mRNA expression was normalised to Oligreen as a measure of total cDNA. QPCR primers were designed using the *Mus musculus* genome and can be found in Table 1. QPCR primers for the mRNA transcripts of adult myosin heavy chain isoforms (*Myh1, Myh2, Myh4, Myh7*), *Asns*, *Atf5*, *Cebpg*, *Phgdh, Psat1*, *Gadd45a* (also known as *Ddit1*) and *Ddit3* (also known as C/EBP Homologous Protein (CHOP)) are reported elsewhere ^29,30^.

**Table 1:** Forward and reverse QPCR primers for the murine genes.

Gene	Forward (5′ → 3′)	Reverse (5′ → 3′)
*Arg2*	ACCACAGCCTGGCAATAGGT	TCAACCCAGATGACACAGAGATCT
*Sars*	TTTGAGAAGATTGAGCAGTTTGTGTA	CGGTGGCGATCATCTCATC
*Slc3a2*	GAGGACAGGCTTTTGATTGCA	TCGCTGGTGGATTCAAGTATGT

### Muscle sectioning and succinate dehydrogenase (SDH) staining

TA muscle was mounted with OCT at below − 20 °C and then sectioned using a Cryostat (Bright instrument: OTF5000-001/D/MR, Serial Number: 065014/5668). Sections (10 µm) were taken after sectioning approximately 100 µm from the transverse cut into the TA muscle. Six sections were collected per slide (Superfrost Plus, Thermo Scientific) and four slides were collected per muscle. Slides were stored at − 20 °C until histochemical analysis was performed. SDH staining was carried out by submerging slides in a SDH incubation solution (saturated disodium succinate, 50 mM Tris–HCl (pH 7.4), 1 mg/ml Nitro blue tetrazolium, 0.02 mM MgCl_2_) and incubated at 37 °C for 1 h. Slides were washed in distilled water for 1 min, followed by 50% ethanol, 70% ethanol and 100% ethanol. Slides were then mounted with Omnimount mounting medium (HS-110, National diagnostics) and left to air-dry in the fume hood overnight. Two or three representative fields of view were captured per muscle section at 10 × magnification and the average minimal feret’s diameters (µm) and fibre areas (µm^2^) determined for 15 randomly selected ‘dark/intermediate (oxidative)’ and ‘light (glycolytic)’ muscle fibres per field of view. Proportions were determined (as a %) relative to the total number of muscle fibres per field of view.

### Statistical analyses

All data are presented as means ± standard error of the mean (SEM). All statistical analyses were performed in Genstat (19^th^ edition), with *P* < 0.05 considered statistically significant. Paired Student’s *t*-tests were used to compare muscle weight and gene expression data from experimental (TA injected with AAV-GOI) and contralateral control (TA injected with AAV-*Gfp*) legs of the same animals. For the AAV-*Atf5* ± *Cebpg* study, a general two-way (AAV × time for muscle weights) or unbalanced two-way (AAV-*Atf5* × AAV-*Cebpg* for gene expression) ANOVA was used, blocking for ‘animal’, followed by post-hoc Bonferroni multiple comparison tests as appropriate (*P* < 0.05 for ANOVA). Minimal feret’s diameters, fibre areas and fibre type proportions were analysed by unpaired *t*-tests.

## Supplementary Information

Supplementary Information.

## Data Availability

The datasets generated and/or analysed during the current study are available from the corresponding author on reasonable request.
