# Supplementary material for: Effect of AAV-mediated overexpression of ATF5 and downstream targets of an integrated stress response in murine skeletal muscle
Source: Sci Rep. 2021 Oct 5;11:19796. doi: 10.1038/s41598-021-99432-4 (PMC8492641; doi:10.1038/s41598-021-99432-4)
Supplement: Supplementary file 1 — Supplementary Information. [file 41598_2021_99432_MOESM1_ESM.pdf]

Effect of AAV-mediated overexpression of ATF5 and downstream targets of an integrated stress response in murine skeletal muscle

Madelaine C. Brearley-Sholto[1,3], David M. Loczenski-Brown[1,4], Sarah Jones[1,5], Zoe Daniel[1], Francis J.P. Ebling[2], Tim Parr[1] and John M. Brameld[1]*

[1]School of Biosciences, University of Nottingham, Sutton Bonington Campus, LE12 5RD, UK

[2]School of Life Sciences, University of Nottingham Medical School, Nottingham, NG7 2UH, UK

[3]Current address: Departments of Biological Chemistry and Medicine, University of California, Los Angeles, California, United States of America

[4]Current address: Sygnature Discovery Limited, BioCity, Nottingham, Nottinghamshire, NG1 1GR, UK

[5]Current address: School of Psychology, University of Nottingham, University Park, NG7 2RD, UK

*Corresponding author: John Brameld, email: john.brameld@nottingham.ac.uk

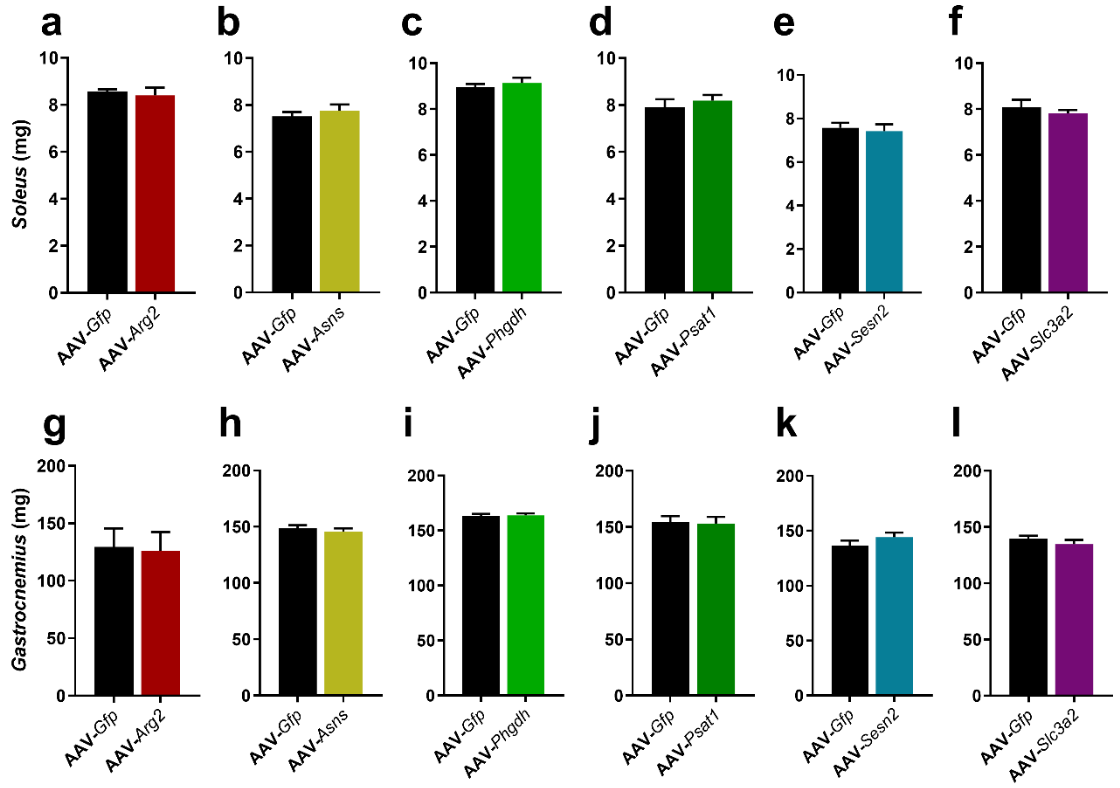

Supplementary Figure 1. Effect of AAV-mediated overexpression of genes associated with an integrated stress response (ISR) on absolute muscle weights

Adeno-Associated Virus 1 (AAV) particles containing a gene of interest (GOI) were injected into mouse *Tibialis Anterior* (TA) muscle and a *Gfp* control sequence alone (AAV-*Gfp*) into the contralateral TA, then maintained in their home cage for 28 days. GOI included Arginase-2 (AAV-*Arg2*), Asparagine synthetase (AAV-*Asns*), Phosphoglycerate Dehydrogenase (AAV-*Phgdh*), Phosphoserine Aminotransferase-1 (AAV-*Psat1*), Sestrin-2 (AAV-*Sesn2*) and Solute Carrier family 3 member 2 (AAV-*Slc3a2*). Data shown as Means (n=8, except AAV-*Sesn2* n=10) ± SEM for absolute *Soleus* (a-f) and *Gastrocnemius* (EDL) (g-l) muscle weights.

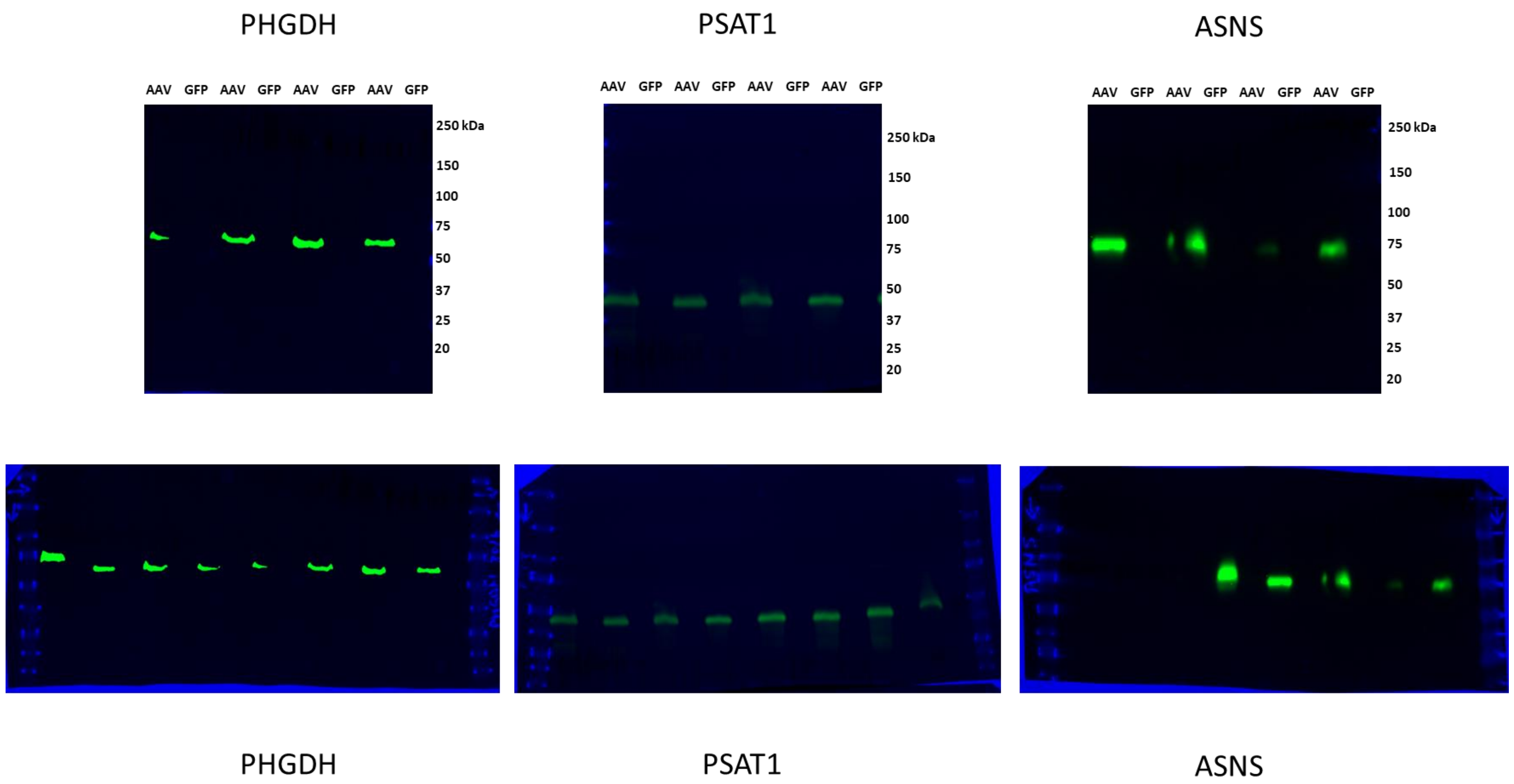

Supplementary Figure 2. Effect of AAV-mediated overexpression on protein levels for PHGDH, PSAT1 and ASNS determined by western blot analyses. Adeno-Associated Virus 1 (AAV) particles containing Phosphoglycerate Dehydrogenase (PHGDH), Phosphoserine Aminotransferase-1 (PSAT1) or Asparagine synthetase (ASNS) were injected into mouse *Tibialis Anterior* (TA) muscle and a GFP control sequence alone (GFP) into the contralateral TA, then

maintained in their home cage for 28 days. Blots representing 4 mice per gene are shown at top, along with molecular weights, but quantification of the fold-changes was not possible due to the lack of bands in the control (GFP) legs. Images of the full blots are shown below. Unfortunately, determination of protein levels for the other genes (*Arg2*, *Sesn2*, *Slc3a2*, *Atf5*, *Cebpg* and *Atf5+Cebpg*) was not possible due to the TA muscles having already been used for histological fibre type analyses.

Supplementary Table 1. Accession numbers and Gene names for sequences with highest % identity to the primers used for QPCR analyses

Gene of Interest	Accession number(s) for sequence(s)	Name of sequence(s) identified as containing the QPCR primers by Blast	% Identity
Arg2	NM_009705.3	Mus musculus arginase type II (Arg2), mRNA	100%
Asns	NM_012055.3	Mus musculus asparagine synthetase (Asns), mRNA	100%
Atf5	NM_030693.2	Mus musculus activating transcription factor 5 (Atf5), transcript variant 1, mRNA	100%
Cebpg	NM_009884.3	Mus musculus CCAAT/enhancer binding protein (C/EBP), gamma (Cebpg), mRNA	100%
Ddit3	NM_007837.4<br>NM_001290183.1	Mus musculus DNA-damage inducible transcript 3 (Ddit3), transcript variant 1, mRNA<br>Mus musculus DNA-damage inducible transcript 3 (Ddit3), transcript variant 2, mRNA	100%<br>100%
Gadd45a	NM_007836.1	Mus musculus growth arrest and DNA-damage-inducible 45 alpha (Gadd45a), mRNA	100%
Idh2	NM_173011.2	Mus musculus isocitrate dehydrogenase 2 (NADP+), mitochondrial (Idh2), nuclear gene encoding mitochondrial protein, mRNA	100%
Phgdh	NM_016966.3	Mus musculus 3-phosphoglycerate dehydrogenase (Phgdh), mRNA	100%
Psat1	NM_177420.2<br>NM_001205339.1	Mus musculus phosphoserine aminotransferase 1 (Psat1), transcript variant 1, mRNA<br>Mus musculus phosphoserine aminotransferase 1 (Psat1), transcript variant 2, mRNA	100%<br>100%
Sars	NM_011319.3<br>NM_001204979.1	Mus musculus seryl-aminoacyl-tRNA synthetase (Sars), transcript variant 1, mRNA<br>Mus musculus seryl-aminoacyl-tRNA synthetase (Sars), transcript variant 2, mRNA	100%<br>100%
Sesn2	NM_144907.1	Mus musculus sestrin 2 (Sesn2), mRNA	100%
Slc3a2	M_001161413.1<br>NM_008577.4	Mus musculus solute carrier family 3 (activators of dibasic and neutral amino acid transport), member 2 (Slc3a2), transcript variant 1, mRNA<br>Mus musculus solute carrier family 3 (activators of dibasic and neutral amino acid transport), member 2 (Slc3a2), transcript variant 2, mRNA	100%<br>100%
